# Experimental susceptibility of *Macrobrachium rosenbergii* (de Man) subadults and adults to infectious myonecrosis virus (IMNV)

**DOI:** 10.1007/s11259-025-11045-8

**Published:** 2026-01-31

**Authors:** Scarlatt Paloma Alves da Silva, Fernando Leandro dos Santos, Suzianny Maria Bezerra Cabral da Silva

**Affiliations:** 1 Department of Fisheries and Aquaculture, Universidade Federal Rural de Pernambuco, Recife, Brazil; 2 Department of Veterinary Medicine, Universidade Federal Rural de Pernambuco, Recife, Brazil

**Keywords:** Giant freshwater prawn, Virus, Nested-PCR, Histopathology

## Abstract

Infectious myonecrosis virus (IMNV) is considered one of the pathogens that most affect the production of *Penaeus vannamei*, causing economic losses estimated at billions of dollars. *P. vannamei* rearing is expanding to inland waters, which are commonly used for the farming of *Macrobrachium rosenbergii*, increasing the probability of exposure of this freshwater prawn to viruses often reported in penaeids, including IMNV. Therefore, the aim of the present study was to investigate the susceptibility of *M. rosenbergii* to IMNV through an intramuscular challenge. *M. rosenbergii* subadults and adults were inoculated intramuscularly with IMNV obtained from the abdominal muscle tissue of naturally infected *P. vannamei*. The viral challenge lasted 21 days. All samples had positive nested-PCR results as well as clinical signs and histopathological findings typical of the virus, such as hemocyte infiltration and muscle sinus dilation. This is the first study to demonstrate the susceptibility of *M. rosenbergii* subadults and adults to IMNV. Although no massive mortalities were detected in the challenged prawns, the presence of *M. rosenbergii* as a viral source in the cultivation environment should not be underestimated.

## Introduction

The production of farmed crustaceans reached a total of 11.2 million tons in 2020, corresponding in monetary terms to US$81.5 billion and approximately 13% of all aquiculture production for human consumption (FAO, 2022). Among species produced in shrimp farming activities, the giant freshwater prawn, *Macrobrachium rosenbergii*, is the fifth most widely farmed crustation in the world, with global production of 294 thousand tons in 2020 (FAO, 2021), to which Brazil contributed an estimated 150 thousand tons (Valenti et al. 2021).

Although *M. rosenbergii* is an important source of aquicultural income, economic losses, especially those caused by infectious diseases, result in fluctuations in production (Bonami and Sri Widada 2011; Chen et al. 2021). There are frequent reports of the incidence of viruses, particularly white tail disease (WTD) or white muscle disease (WMD), which is the greatest threat to the viability of *M. rosenbergii* production due to the combination of *M. rosenbergii* nodavirus (MrNV) and extra small virus (XSV) (Hooper et al. 2022). The main clinical signs of WTD are the whitish coloration of the muscles, which begins in the abdomen and affects the entire musculature of the animal in more advanced stages, as well as lethargy and anorexia (Bonami and Sri Widada 2011).

Besides *M. rosenbergii*, infection by MrNV has been reported in *Penaeus vannamei* farmed in Asia (Senapin et al. 2012). In the study cited, after the screening of samples negative for infectious myonecrosis virus (IMNV) and *Penaeus vannamei* nodavirus (PvNV) via real-time polymerase chain reaction (RT-PCR), a reassessment was performed of this material from grow-out farms with high mortality rates and reports of animals with muscle opacity as well as positive RT-PCR results for MrNV; moreover, infectiousness was demonstrated when *P. vannamei* was fed chopped tissue from *M. rosenbergii* infected by MrNV at low temperatures and salinity of the cultivation water (Senapin et al. 2012). In another study, Senapin et al. (2013) reported coinfection by IMNV and MrNV in *P. vannamei* subadults farmed in Indonesia detected by nested RT-PCR and immunohistochemical analysis, with the occurrence of muscle lesions in the coinfected animals.

*P. vannamei* is the only marine shrimp farmed in Brazil (IBGE, 2021), and its farming in brackish and freshwater environments as it expands inland (Pimentel et al. 2022), increases the probability of approaching areas of *M. rosenbergii* production and exposing this species to other viruses, including IMNV, which is considered endemic in northeast Brazil (Prasad et al. 2017). IMN results in muscular opacity of the abdominal segments due to tissue necrosis, and is responsible for 40% to 80% of mortality in shrimp farming, causing estimated US$ 440 million in losses in Brazil between 2002 and 2005 (Andrade et al. 2007). Therefore, the aim of the present study was to investigate the susceptibility of *M. rosenbergii* to IMNV through a viral challenge.

## Materials and methods

### Preparation of viral inoculum

The viral inoculum used came from a strain endemic to Northeast Brazil, obtained from a naturally infected animal during an outbreak on a commercial farm. The presence of IMNV in the tissue used for the viral inoculum (100 g) was confirmed by nested PCR (Poulos and Lightner 2006) and the absence of infection by Taura Syndrome Virus (TSV), White Spot Syndrome Virus (WSSV), Infectious Hypodermal and Hematopoietic Necrosis Virus (IHHNV), and Necrotizing Hepatopancreatitis Bacterium (NHPB) was confirmed by nested PCR, via IQ2000TM kits, according to the manufacturer’s intructions (Farming IntelliGene Tech. Corp., Taiwan) (data not shown). The protocol for preparing the IMNV viral inoculum was carried out according to Silva et al. (2015) and, for this purpose, the tissue was then homogenized in 300 mL of 2% sterile saline solution (1:3, v: v). The homogenized material was successively filtered (300, 210 and 70 μm) to obtain the inoculum, which was diluted at 1:10. The viral load was determined using the method described by Silva et al. (2011) (1.1 × 10³ copies of IMNV/µg of RNA), followed by storage at − 80 °C until use in the viral. challenge.

### Experimental conditions

Fifty-four healthy individuals of *M. rosenbergii* were used in this experiment, including subadults (5–15 g) and adults (16–26 g) (Valenti 1998). The adults were randomly distributed, with no separation between males and females for the experiment. All specimens were from the Johei Koike Mainland Aquiculture Station of Universidade Federal Rural de Pernambuco (UFRPE). The prawns were distributed according to weight and kept in experimental units with 15 L of useful volume at a density of one animal per 5 L at 28 °C in freshwater. The animals were fed a commercial ration (35% of crude protein; Guabitech Active 35, Brazil) corresponding to 3% of the biomass twice per day, as described by D’Abramo and Sheen (1994).

The water used in the system was previously filtered (30 μm) and treated with chlorine (30 ppm). Alkalinity and hardness were maintained above 50 mg of CaCO_3_/L by the addition of sodium carbonate. The volume of the experimental units was renewed daily at a rate of 20% of the total volume. Daily cleaning of the experimental units was performed through siphoning, with the replacement of the volume of water lost during the procedure.

Temperature, pH, and oxygen were monitored twice per day. Water samples were collected weekly for the analysis of nitrite, nitrate, ammoniacal nitrogen, alkaline and hardness using a commercial colorimetric kit (Alcon Labcon, Camboriú, Brazil) to ensure that these parameters were within the ideal limits for carids proposed by New et al. (2010).

All experimental conditions were conducted in accordance with ethical and experimental precepts, authorized by the Ethics Committee on the Use of Animals (CEUA) of the Universidade Federal Rural de Pernambuco (UFRPE).

### Viral challenge

The susceptibility of *M. rosenbergii* subadults and adults to IMNV was determined via an intramuscular injection, following the method described by Silva et al. (2015). The treatments were (1) *M. rosenbergii* subadults experimentally challenged with IMNV, (2) *M. rosenbergii* adults experimentally challenged with IMNV, (3) unchallenged *M. rosenbergii* subadults (control group 1), and (4) unchallenged *M. rosenbergii* adults (control group 2).

Treatments 1 and 2 comprised six repetitions with three animals each, whereas treatments 3 and 4 (control groups 1 and 2) comprised three repetitions with three animals each. All animals in treatments 1 and 2 were challenged with 100 µL of viral inoculum (dilution: 1:10) using an insulin syringe and an intramuscular injection of the third abdominal segment, as described by Silva et al. (2015). In the negative control group, the animals were injected with 100 µL of 0.85% sterile saline solution.

Throughout the challenge, the animals were monitored daily for the observation of clinical signs of IMNV and mortality. The experiment lasted 21 days, during which time dead animals were collected and stored at − 80 °C for subsequent nested-PCR analysis. Survivors were sacrificed on Day 21 and also stored at − 80 °C for subsequent molecular analysis.

### Molecular analysis

#### RNA extraction and RT-PCR

Total RNA was extracted from the muscle (60 mg) of the second abdominal segment in 1 mL of TRIzol (Invitrogen, USA), following the manufacturer’s instructions. After extraction, RNA was qualitatively and quantitatively assessed via spectrophotometry at 260 and 280 ηm (NanoVue Plus™ spectrophotometer, GE Healthcare, USA) and stored at − 80 °C. RT-PCR was then performed using the Improm-II™ Reverse Transcription System (Promega, Madison, WI, USA) in a final volume of 20 µL, with 300 ηg/µL of total RNA and 0.5 µg of oligo(dT)_15_, following the manufacturer’s instructions. cDNA was stored at − 20 °C until the nested-PCR analysis.

#### Nested PCR

Infection by IMNV in *M. rosenbergii* subadults and adults was determined by nested PCR using the specific primers described by Poulos and Lightner (2006). This is the confirmatory diagnosis recommended for IMNV by the World Organization for Animal Health (OIE, 2023). The reaction conditions and thermal cycles were those described by Poulos and Lightner (2006). The products of the first (328 bp) and second (139 bp) PCR were submitted to electrophoresis in 2% agarose gel stained with ethidium bromide, and fragment size was estimated using a 100-bp molecular weight marker (Invitrogen, USA). In each set of nested PCR reactions, a positive control was used, with *P. vannamei* tissues positive for IMNV, as well as a negative control, replacing the cDNA with ultrapure water (Sigma, USA).

### Histopathological analysis

All animals in the different treatments were submitted to histopathological analysis for the identification of lesions suggestive of IMNV. For such, the prawns were fixed in a Davidson’s alcohol-formalin-acetic acid solution for 72 h and transferred to a 70% ethanol solution. The samples were then dehydrated, clarified, embedded in paraffin, cut to a thickness of 3 to 5 μm and stained with hematoxylin and eosin, as described by Lightner (1996).

All slides were examined under an optical microscope for the determination of coagulative necrosis among the muscle fibers of the abdominal segments, hemocyte infiltration among the muscle fibers, and the occurrence of cytoplasmic inclusion bodies (Tang et al. 2005).

### Statistical analysis

As normal distribution and equality of variances were confirmed by the Shapiro-Wilk test and Bartlett’s test, respectively, analysis of variance (ANOVA) and the Student’s t-test were used to determine statistically significant differences between treatments (*p* ≤ 0.05). The data from the nested-PCR analysis and data on mortality were submitted to descriptive statistics. The cumulative survival probability for each group was estimated using the Kaplan-Meier method. Curves were plotted to visualize the mortality dynamics of both groups over time. Confidence intervals of 95% were calculated and plotted to indicate the precision of the estimates. To determine whether the observed difference in survival curves was statistically significant, the log-rank test was used. This test compares the survival distribution between groups, with the null hypothesis (H0) that there is no difference in mortality between adults and subadults. A p-value less than 0.05 was considered statistically significant. All data were analyzed using R software (version 4.5.1) and the survival and survminer packages.

## Results and discussion

**Fig. 1:**
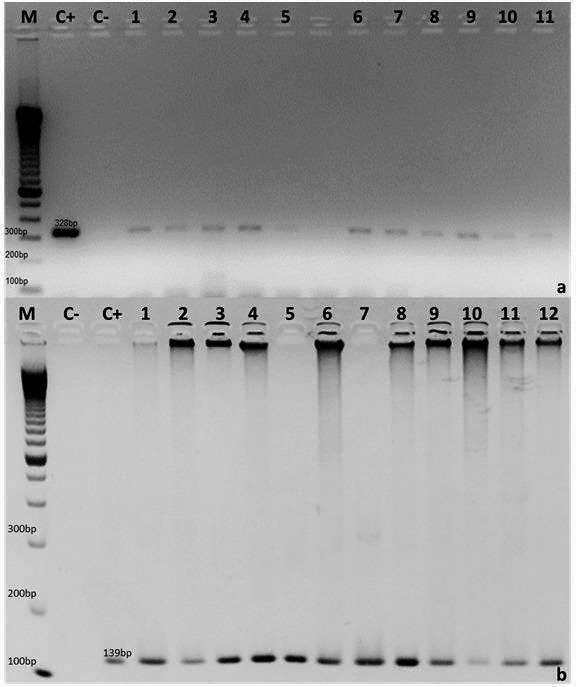
Detection of IMNV in *Macrobrachium rosenbergii* subadults and adults experimentally infected with intramuscular injection. M: 100-bp molecular weight marker (Invitrogen, USA); C+: positive control; C−: negative control (ultrapure water); (**a**) 1 to 5: samples of *M. rosenbergii* subadults positive for IMNV; 6 to 11: samples of *M. rosenbergii* adults positive for IMNV; (**b**) 1 to 6: samples of *M. rosenbergii* subadults positive for IMNV; 7 to 12: samples of *M. rosenbergii* adults positive for IMNV

All samples of *M. rosenbergii* subadults and adults challenged with IMNV (1.1 × 10³ copies of IMNV/µg of RNA) had positive nested-PCR results (Fig. 1), showing clinical signs of infection such as multifocal opacity in the muscles of the abdominal segment, lethargy, and a reduction in food intake. No massive mortalities occurred during the period of the experiment (Table 1).

**Table 1:** Cumulative mortality in different treatments of *M. rosenbergii* challenged with IMNV

Treatment	Number of individuals	Cumulative mortality				
		Day 1	Day 7	Day 14	Day 21	Day 22^*^
1	18	0	2	8	1	7
2	18	0	2	4	3	9
3	9	0	3	3	0	3
4	9	0	2	0	2	5

^*^Sacrificed animals. Treatment: 1- *M. rosenbergii* subadults experimentally challenged with IMNV; 2- *M. rosenbergii* adults experimentally challenged with IMNV; 3- Unchallenged *M. rosenbergii* subadults (control group 1); 4- Unchallenged *M. rosenbergii* adults (control group 2)

In the first PCR, nine animals were positive in treatment 1 (*M. rosenbergii* subadults experimentally challenged with IMNV) and 13 animals were positive in treatment 2 (*M. rosenbergii* adults experimentally challenged with IMNV) (Table 2). All samples were positive in the second PCR (Table 2). All animals in treatments 3 and 4 (control groups 1 and 2) had negative results in both nested-PCR analyses (Table 2) and had no signs of infection.

**Table 2:** Number of individuals infected in four different treatments of *M. rosenbergii* challenged with IMNV

Treatment	Number analyzed	Positive 1st PCR	Positive 2nd PCR
1- *M. rosenbergii* subadults experimentally challenged with IMNV	18	9	18
2- *M. rosenbergii* adults experimentally challenged with IMNV	18	13	18
3- Unchallenged *M. rosenbergii* subadults (control group 1)	9	0	0
4- Unchallenged *M. rosenbergii* adults (control group 2)	9	0	0

IMNV has been detected in other species. Investigating the susceptibility of juveniles of *P. vannamei*, *L. stylirostris*, and *Penaeus monodon* for four weeks through an intramuscular challenge of an inoculum containing purified virions, Tang et al. (2005) demonstrated infection by IMNV on the 14th day post-inoculation (dpi) via in situ hybridization. The authors also found clinical signs typical of IMNV (whitish lesions in the muscles of the abdomen and tail) in all challenged individuals of *P. vannamei* on the sixth dpi, whereas such lesions were observed in *L. stylirostris* only on the 13th day and no clinical signs were found in *P. monodon*. In the present study, clinical signs (muscle opacity) were found in *M. rosenbergii* subadults and adults experimentally infected with IMNV.

Infection by IMNV was also found in wild breeders of *P. monodon* caught in the northeastern Indian Ocean. Among the six lots analyzed using nested-PCR, only two had infected animals, with a total of six positive samples among the 90 analyzed in these lots, with no detection of the basophilic cytoplasmic inclusion bodies characteristic of IMNV reported for penaeids (Srisala et al. 2021).

In a study involving the challenge of *Fenneropenaeus merguiensis* and *Penaeus esculentus* through noninvasive procedures that reproduced natural infection routes, such as transmission through water and predation (ingestion of tissue of *P. vannamei* contaminated with IMNV), Gudkovs et al. (2015) performed a set of molecular tests (PCR, sequencing and in situ hybridization) and histological analysis and determined that both species are susceptible to IMNV, with the confirmation of infection and the demonstration of the replication of the virus in both species.

Besides the occurrence of IMNV in diverse species, viral coinfection has also been reported. After the collection of 30 ill specimens of *P. vannamei* (10 ± 2 g) from a farm in the state of Ceará in northeast Brazil, Feijó et al. (2013) found that 12 had positive qPCR results for both IMNV and WSSV, and also described the following histopathological lesions in the animals: (i) hemocyte infiltration and coagulative necrosis (16/30 animals); (ii) hypertrophy of lymphoid and spheroid organs (9/30 animals); and (iii) ectopic spheroids, mainly close to the antennal gland and in the skeletal muscle (6/30 animals).

Assessing the impact of infectious hypodermal and hematopoietic necrosis virus (IHHNV) and IMNV through an oral challenge in wild juveniles of *Farfantepenaeus subtilis* (2.56 ± 0.44 g), Coelho et al. (2009) found that only 10% of the challenged animals were positive for both viruses (determined by PCR). The authors also reported signs of tissue changes in the specimens infected with IMNV, in which all positive animals had low levels of hemocyte infiltration and mild coagulation.

After the screening of 15 specimens of *P. vannamei* with signs of whitish muscles and a suspicion of infection by IMNV collected from out-growth nurseries of eight farms in three provinces of Indonesia, Senapin et al. (2013) found that 10 animals were positive for IMNV and six of these animals were also positive for *Macrobrachium rosenbergii* nodavirus (MrNV). MrNV and extra small virus (XSV) are the etiological agents of white tail disease (WTD) in *Macrobrachium rosenbergii*, the most common symptoms of which are opacity of the abdominal muscle and degeneration of the telson and uropods (Chen et al. 2021).

**Fig. 2:**
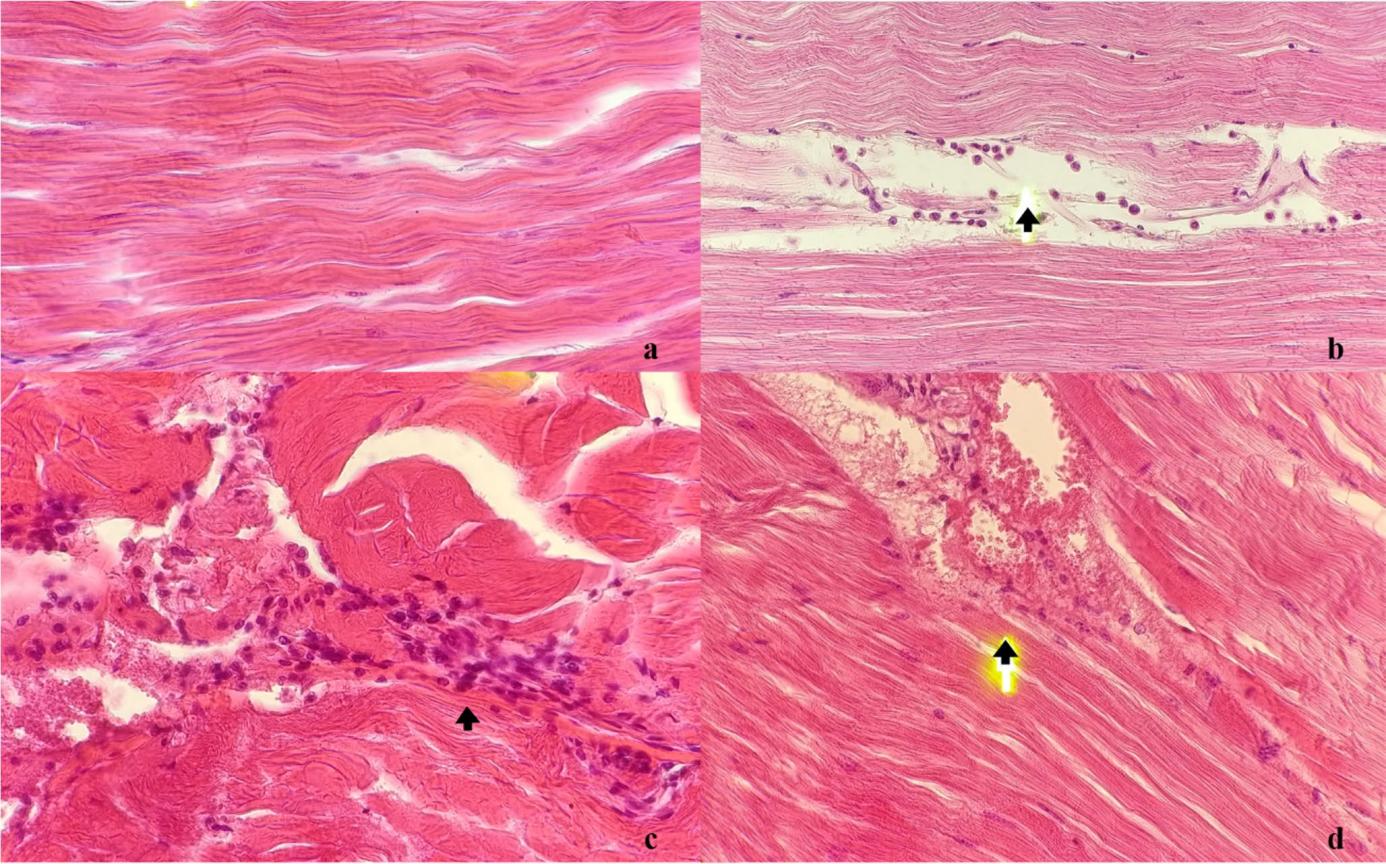
Photomicrographs of *M. rosenbergii* tissue examined for lesions suggestive of IMNV (a, b, c and d): (**a**) Adult from control group with no apparent change in muscle (arrow) in 6th right abdominal segment. HE staining. Magnification: 400×; (**b**) Infected subadult (treatment 1) with discrete hemocyte infiltration among muscle bundles (arrow) in 6th right abdominal segment. HE staining. Magnification: 400×; (**c**) Infected adult (treatment 2) with intense hemocyte infiltration among muscle bundles (arrow) and coagulative necrosis in 1st right abdominal segment. HE staining. Magnification: 400×; (**d**) Infected adult (treatment 2) with dilation in sinus and discrete hemocyte infiltrate (arrow) in 6th right abdominal segment. HE staining. Magnification: 400×

The histopathological analysis revealed hemocyte infiltration (Fig. 2b, c and d) and sinuses dilation in the muscles of the animals experimentally inoculated with IMNV in both treatments 1 and 2 (Fig. 2d). In contrast, no tissue changes were found in the control groups (*M. rosenbergii* subadults and adults inoculated with 0.85% saline solution) (Fig. 2a).

Tang et al. (2005) reported the occurrence of skeletal muscle lesions, including coagulative necrosis, fibrotic inflammation, fibrosis, and cytoplasmic inclusion bodies, when the penaeid species *P. vannamei*, *L. stylirostris*, and *Penaeus monodon* were experimentally challenged with IMNV. These histopathological findings are compatible with those in *M. rosenbergii* subadults and adults in the present study (Fig. 2).

**Fig. 3:**
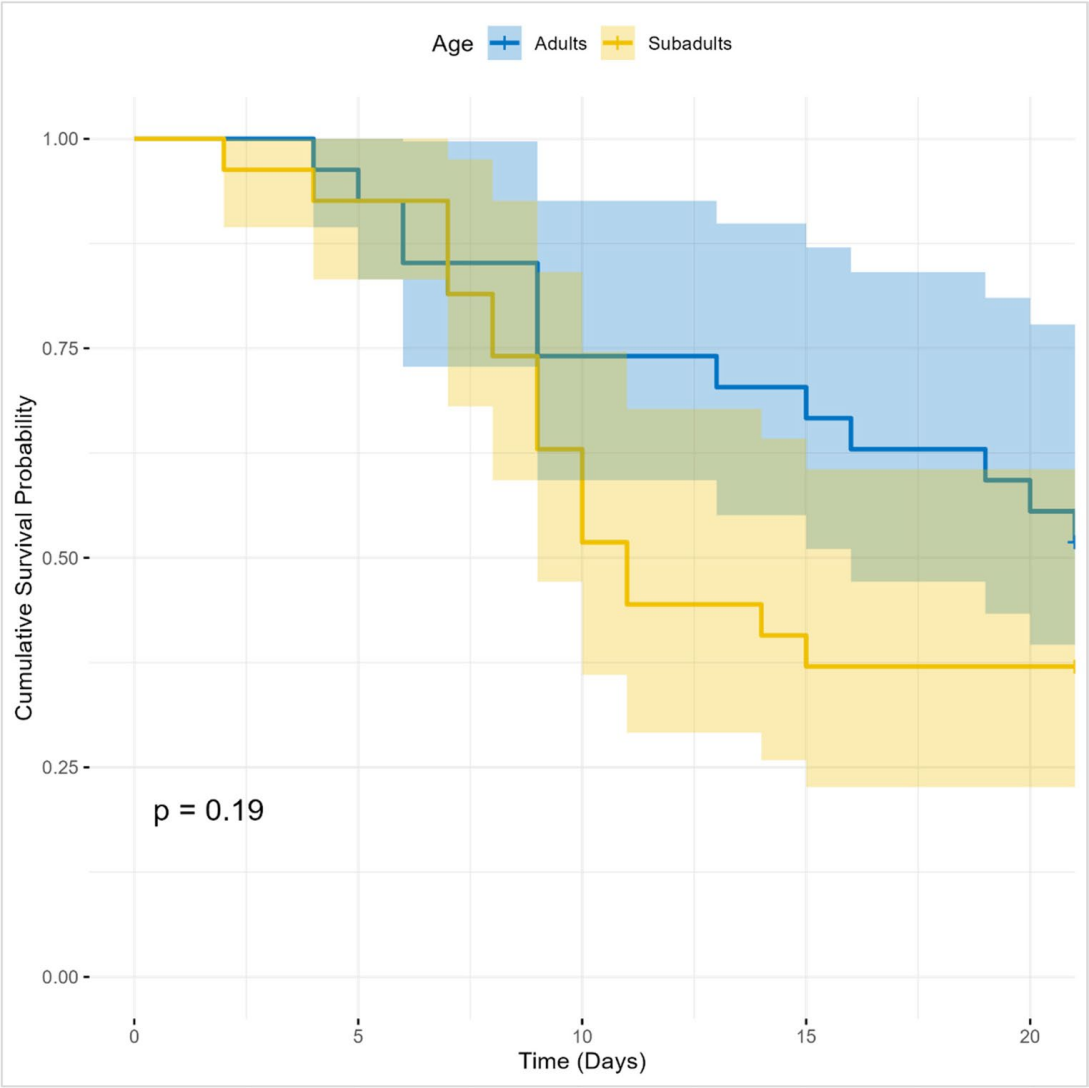
Cumulative survival probability of adults and subadults throughout the experiment

Regarding mortality rates, there is no statistically significant evidence that they differed between the adult and subadult groups (p-value of 0.19) (Fig. 3). Furthermore, although the subadult curve appears to have a higher mortality rate than the adults, this difference may simply be a random variation in the sample and not a true difference in the population.

The combination of data from the nested-PCR and histopathological analyses shows that *M. rosenbergii* is sensitive to IMNV. This is the first study to demonstrate the susceptibility of *M. rosenbergii* subadults and adults to IMNV.

## Conclusions

The present study demonstrated the susceptibility of *M. rosenbergii* subadults and adults to infection by IMNV under experimental conditions. Although no massive mortality occurred during the 21 days of the experiment among the challenged prawns, one should not underestimate the presence of *M. rosenbergii* as a viral source. Future studies are suggested to assess the role of *M. rosenbergii* as a vector in the horizontal transmission of IMNV in *P. vannamei*.

## Data Availability

No datasets were generated or analysed during the current study.
